# MgO-Loaded Magnetic Crab Shell-Derived Biochar for Efficient Synergistic Adsorption of Heavy Metals and Dye: Characterization, Adsorption Performance and Mechanistic Study

**DOI:** 10.3390/nano16030214

**Published:** 2026-02-06

**Authors:** Yangyi Du, Si Wu, Tao Feng, Wenxue Jiang

**Affiliations:** 1 College of Resources and Environmental Engineering, Wuhan University of Science and Technology, Wuhan 430081, China; 2 Hubei Key Laboratory for Efficient Utilization and Agglomeration of Metallurgic Mineral Resources, Wuhan University of Science and Technology, Wuhan 430081, China

**Keywords:** MgO, crab shell, magnetic biochar, heavy metal, Congo Red, simultaneous adsorption

## Abstract

The preparation of highly efficient adsorbents capable of simultaneously removing dyes and heavy metals is of great importance. Crab shell-derived biochar (BC) was successfully modified with magnesium and iron oxides (magnetic MgO@BC) via a simple impregnation–carbonization method. A series of characterizations revealed that magnetic MgO@BC possessed hierarchical porous structure with abundant oxygenated functional groups and good magnetic separability. The results of batch adsorption experiments showed that the actual maximum adsorption capacities of magnetic MgO@BC were 301.06, 1344.11 and 3232.10 mg/g for Cd^2+^, Pb^2+^ and CR, respectively. In addition, the adsorption of Cd^2+^, Pb^2+^, and CR exhibited minimal influence from pH and coexisting ions, except for Cd^2+^ adsorption, which was significantly affected by divalent cations. For Cd^2+^ and Pb^2+^ adsorption, the Langmuir model provided good fits for the adsorption isotherms, whereas CR adsorption was more suitable for the Freundlich model. The adsorption kinetic fitting results indicate that Cd^2+^ adsorption aligned well with the pseudo-first-order model, while Pb^2+^ and CR fitted better with the pseudo-second-order model. Regeneration tests revealed that after four cycles, Cd^2+^, Pb^2+^ and CR still maintained 85.87%, 52.43%, and 96.09% removal efficiencies, respectively. SEM, FTIR, XRD, and XPS results demonstrated that the mechanism for CR adsorption involved π-π interactions, electrostatic attraction, and hydrogen bonding. The adsorption mechanism of heavy metals was primarily governed by ion exchange, cation-π interactions, surface coordination, and coprecipitation mechanisms, where Pb^2+^ exhibited stronger and more preferential adsorption behavior. Binary adsorption experiments confirmed competitive and synergistic effects depending on pollutant pairs. This study offers a novel perspective on the preparation and mechanism of biochar materials for the efficient and synergistic removal of dyes and heavy metals.

## 1. Introduction

Industrial effluents commonly contain persistent contaminants such as organic dyes and heavy metals, both posing serious threats to ecosystems and human health. Azo dyes such as Congo Red (CR) primarily originate from the textile, electroplating, and printing industries, which are toxic and resistant to biodegradation, leading to oxygen depletion, ecological imbalance, and carcinogenic risks [1,2]. Heavy metals like Pb^2+^ and Cd^2+^ are commonly found in wastewater. They are non-biodegradable and bioaccumulative, potentially causing renal damage, neurotoxicity, and cancer [3,4]. Conventional wastewater treatment methods, including coagulation-flocculation, membrane separation, ion exchange, chemical precipitation, advanced oxidation processes (AOPs), and biological treatments, often suffer from high costs, operational complexity, and secondary pollution issues [5]. In contrast, adsorption has emerged as an efficient, low-cost, environmentally friendly, and regenerable technique [6].

Biochar extracted from agricultural waste can effectively eliminate heavy metals and organic pollutants in wastewater through adsorption, owing to its porous structure, large specific surface area, and numerous surface functional groups [7]. Biochar obtained from Opuntia ficus-indica was prepared for the removal of green dyes and metal ions in water [8]. Crab shells represent an abundant seafood waste composed mainly of chitin, protein, and CaCO_3_. The chitin provides functional –OH and –NH_2_ groups favorable for metal ion chelation. Consequently, crab shells serve as low-cost, sustainable feedstock for biochar and composite adsorbents [9,10]. For example, mesoporous activated crab shell biochar demonstrated excellent tetracycline adsorption [11], and the chitin/calcite composite (CCA) extracted from crab shell waste exhibited maximum adsorption capacities of 228.86 mg/g for ciprofloxacin (CIP) and 150.76 mg/g for tetracycline (TC) at 25 °C [10]. Dai et al. prepared calcium-rich biochar (CRB) from crab shells, which exhibited ultrahigh maximum adsorption capacities of 12,502 mg/g for the cationic dye malachite green and 20,317 mg/g for the anionic dye Congo Red [12]. However, pristine crab shell biochar exhibits notable limitations: its adsorption capacity for heavy metals is relatively low, and it lacks recoverability, requiring tedious centrifugation/filtration after adsorption, which restricts its practical application in complex wastewater treatment.

To further enhance the adsorption performance and facilitate efficient recovery, some researchers employ nano-metal oxide modification and magnetic modification to increase its surface area and pore volume [13]. The incorporation of magnesium oxide (MgO) onto the biochar surface introduces abundant Lewis basic sites, which significantly improve the interaction with acidic pollutants including heavy metal ions and dyes [14]. Simultaneously, MgO loading contributes to the development of hierarchical porous structures and increases the specific surface area, thereby providing more accessible active sites for pollutant binding and accelerating the adsorption kinetics [15]. However, MgO-modified biochar often suffers from particle aggregation, which reduces the exposure of active sites. For instance, MgO-loaded fish scale biochar exhibited ultrahigh adsorption of Cu^2+^/Cd^2+^/Pb^2+^ (505.8, 327.2, and 661.2 mg/g) [16]. While incorporation of Fe_3_O_4_ yields magnetically separable adsorbents [17]. Weidner et al. fabricated hybrid metal oxide/biochar composites with magnetic components that effectively synergistically removed As^3+^ and Pb^2+^ [18]. Zhang et al. synthesized MgO template-derived mesoporous magnetic carbons, enabling fast heavy metal ion removal [19]. Ruthiraan et al. prepared magnetic biochar derived from mangosteen peels. The q_max_ values of the material are 45.662 and 46.296 mg/g for Cd^2+^ and methylene blue [20]. Suwunwong, T. et al. prepared magnetic biochar by compositing rice husk-derived biochar with Fe_3_O_4_ nanoparticles, and the resulting material achieved a maximum removal efficiency of 94% for rhodamine 6G when pyrolyzed at 500 °C [21]. However, a limitation of magnetic modification is that the loading of magnetic particles may block the pores of the biochar, leading to a decrease in adsorption capacity.

Notably, few studies have combined MgO modification, magnetic modification, and CaCO_3_-rich crab shell biochar. This composite design is expected to achieve synergistic advantages among the components: CaCO_3_ in crab shells enhances precipitation and ion exchange, MgO provides abundant Lewis basic sites to strengthen the affinity for both dyes and heavy metals, and Fe_3_O_4_ enables magnetic recovery. Therefore, this approach helps to address the inherent limitations of singly modified biochar.

Given the need for adsorbents with high adsorption capacity, magnetic recoverability and the ability to simultaneously remove dyes and metal ions, we synthesized magnetic MgO-loaded crab shell-based biochar (magnetic MgO@BC). This study systematically evaluated the adsorption performance and adsorption models of magnetic MgO@BC in both single pollution systems (Cd^2+^, Pb^2+^ and CR) and binary-pollutant systems (CR–Pb^2+^, CR–Cd^2+^ and Pb^2+^–Cd^2+^). The adsorption mechanism and regenerability were also thoroughly explored. This work provides valuable insights into the modification of biochar materials and the synergistic removal of dyes and heavy metals in complex wastewater remediation.

## 2. Materials and Methods

### 2.1. Materials

Crab shells were purchased from Bozhou Qiaoshengtang Biotechnology Co., Ltd. (Bozhou, China). Magnesium chloride hexahydrate (MgCl_2_·6H_2_O), ferrous chloride tetrahydrate (FeCl_2_·4H_2_O), Congo Red (industrial grade), lead nitrate (Pb(NO_3_)_2_), cadmium nitrate tetrahydrate (Cd(NO_3_)_2_·4H_2_O), hydrochloric acid (HCl), sodium chloride (NaCl), potassium chloride (KCl), anhydrous calcium chloride (CaCl_2_), sodium hydroxide (NaOH), anhydrous sodium sulfate (Na_2_SO_4_), and anhydrous sodium carbonate (Na_2_CO_3_) were all supplied by Sinopharm Chemical Reagent Co., Ltd. (Shanghai, China). Standard solutions of Pb^2+^ and Cd^2+^ (1000 μg/mL) were purchased from the National Center for Nonferrous Metals and Electronic Materials Testing (Beijing, China). All reagents employed in this work were of analytical grade and were used directly without additional purification, unless specified otherwise.

### 2.2. Preparation of Magnetic MgO@BC

The purchased crab shells were first thoroughly washed and oven-dried at 60 °C. After drying, they were ground using a planetary ball mill and passed through a 200-mesh sieve to obtain fine powder. The sieved crab shell powder was then subjected to pre-carbonization in a tubular furnace at 300 °C under a nitrogen atmosphere, with a heating and cooling rate of 10 °C/min. After that, the pre-carbonized crab shell biochar was immersed in 0.04 M MgCl_2_·6H_2_O and 0.02 M FeCl_2_·4H_2_O solution [22], and the pH was adjusted to 9 with 0.1M NaOH. After impregnation for 8 h, the material was dried and subsequently subjected to carbonization at 700 °C under a nitrogen atmosphere in a tubular furnace, with a heating and cooling rate of 10 °C/min. The exploration of the material preparation conditions is detailed in Figures S1 and S2 of the Supplementary Materials. The obtained composite was then washed repeatedly with deionized water until the supernatant reached neutral pH. The final product, denoted as magnetic MgO@BC, was collected and stored for further characterization and application.

### 2.3. Characterizations

Fourier transform infrared spectroscopy (FTIR, Nicolet iS20, Thermo Fisher Scientific, Waltham, MA, USA) was employed to identify surface functional groups in the range of 4000–400 cm^−1^ using the KBr pellet method. Scanning electron microscopy with Energy-Dispersive X-ray Spectroscopy (SEM–EDS, MIRA LMS, TESCAN, Brno, Czech Republic) was used to observe surface morphology and analyze elemental distribution. Samples were gold-coated using a Quorum SC7620 sputter coater. X-ray diffraction (XRD, SmartLab SE, Rigaku, Suita, Japan) was conducted to determine the crystalline phase composition using Cu Kα radiation over a 2θ range of 5–90° at a scanning rate of 5°/min. X-ray photoelectron spectroscopy (XPS, K-Alpha, Thermo Scientific, Waltham, MA, USA) was used to analyze surface elemental composition and chemical states of C, O, N, Mg, and Fe. Measurements were performed using Al Kα radiation (1486.6 eV) with a 400 μm spot size at 12 kV. Thermogravimetric–Differential scanning calorimetry (TG–DSC, SDT Q600, TA Instruments, New Castle, DE, USA) was carried out to assess thermal stability and composition. Samples were heated from room temperature to 800 °C at 10 °C/min under N_2_ atmosphere. Nitrogen adsorption–desorption measurements were performed using a BET surface area analyzer (Micromeritics ASAP 2460, Micromeritics Instrument Corporation, Norcross, GA, USA) to characterize the textural properties. The specific surface area was determined by the BET method, and the pore size distribution was obtained from the desorption curve according to the BJH model. Vibrating sample magnetometry (VSM) measurements were carried out to characterize the magnetic behavior of the composites.

### 2.4. Adsorption Experiments

#### 2.4.1. Adsorption Performance in Single-Pollutant Systems

To investigate the adsorption performance of magnetic MgO@BC, a series of batch experiments were performed to evaluate the effects of initial concentration, adsorbent dosage, pH, temperature, and coexisting ions on the removal of Cd^2+^, Pb^2+^ and Congo Red (CR). All experiments were conducted in triplicate. The samples were placed in a thermostatic shaker at 25 °C and 180 rpm unless otherwise stated. In the adsorption experiments, the counter-ion for both Pb(II) and Cd(II) was nitrate (NO_3_^−^).

For the initial concentration experiments, 15 mg and 5 mg of adsorbent was added, respectively, to 50 mL Cd^2+^ and Pb^2+^ solutions with concentrations of 10, 20, 30, 60, 80, and 100 mg/L. 25 mg of adsorbent was added to 50 mL of CR solutions with initial concentrations of 100, 200, 300, 500, 600, and 1000 mg/L.

To evaluate the influence of adsorbent dosage, 50 mL of Cd^2+^ (50 mg/L), Pb^2+^ (50 mg/L), and CR (500 mg/L) solutions were treated with varying adsorbent dosages ranging from 0.1 to 0.5 g/L.

For the pH effect study, the initial pH of Cd^2+^ and Pb^2+^ solutions (50 mg/L) were adjusted from pH 3 to 7, with respective dosages of 0.3 g/L and 0.1 g/L. While CR solutions (500 mg/L) were adjusted from 3 to 11 with 0.3 g/L dosage.

Temperature-dependent adsorption behavior was examined at 5, 15, 25, and 35 °C for Cd^2+^ (50 mg/L, 0.3 g/L dosage), Pb^2+^ (50 mg/L, 0.1 g/L dosage), and CR (500 mg/L, 0.3 g/L dosage).

The influence of coexisting ions was investigated using NaCl, KCl, MgCl_2_, and CaCl_2_ (2500 mg/L) for Cd^2+^ (50 mg/L, 0.3 g/L dosage) and Pb^2+^ (50 mg/L, 0.1 g/L dosage), and NaOH, NaCl, Na_2_SO_4_, and Na_2_CO_3_ (2500 mg/L) for CR (200 mg/L, 0.3 g/L dosage).

In addition, adsorption kinetics and isotherm models were applied to fit the experimental data for all three pollutants. The concentration of CR was determined using a UV-Vis spectrophotometer (TU-1810, Purkinje General, Beijing, China), while the concentrations of Cd^2+^ and Pb^2+^ were measured using a flame atomic absorption spectrometer (novAA 350, Analytik Jena AG, Jena, Germany). These systematic studies provide insights into the adsorption behavior and potential mechanisms of magnetic MgO@BC in removing both organic dyes and heavy metal ions.

#### 2.4.2. Regeneration Experiments

The reusability of magnetic MgO@BC was assessed through regeneration experiments. For pollutant-loaded samples, thermal regeneration was carried out in a tubular furnace under a nitrogen atmosphere at 700 °C for 30 min, with both heating and cooling rates maintained at 10 °C/min. All experiments were performed in triplicate.

#### 2.4.3. Adsorption Performance in Binary-Pollutant Systems

The co-adsorption behavior of CR–Pb^2+^, CR–Cd^2+^, and Cd^2+^–Pb^2+^ pairs was evaluated through binary adsorption tests. For the study of binary systems, the concentration ratios of 50 mg/L with 50 mg/L, 100 mg/L with 50 mg/L, 100 mg/L with 100 mg/L, and 50 mg/L with 100 mg/L were explored, separately. All experiments were conducted in triplicate and performed in a thermostatic shaker at 25 °C and 180 rpm.

## 3. Results and Discussion

### 3.1. Characterization of Magnetic MgO@BC

**Figure 1:**
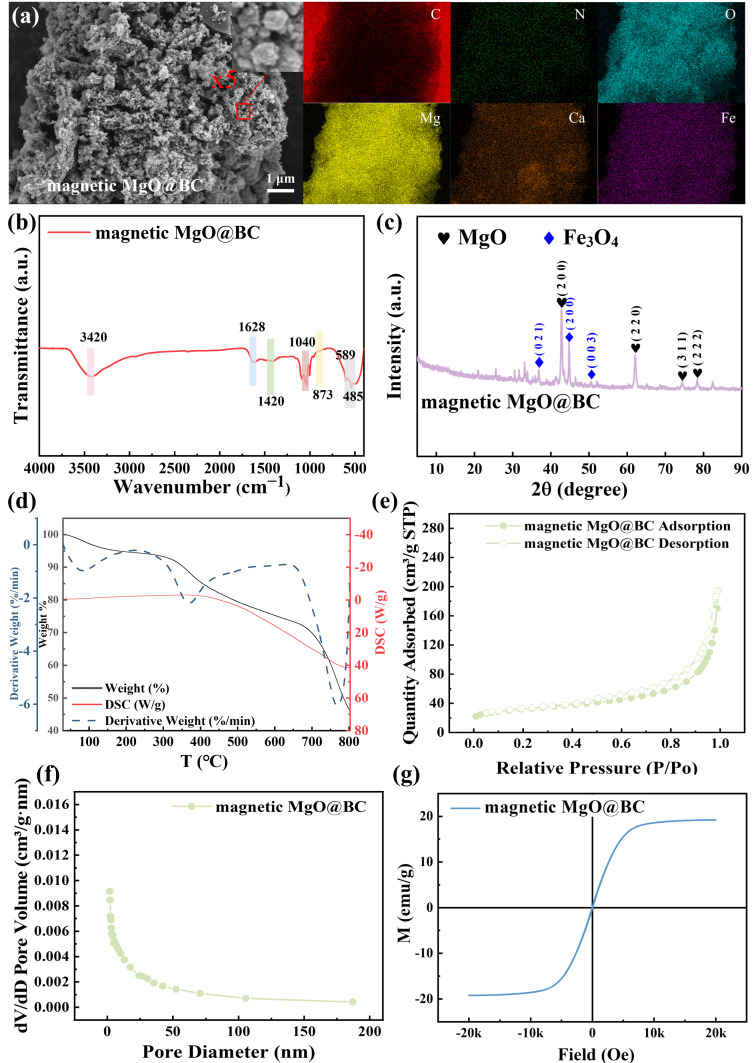
(**a**) The SEM images and the elemental mappings, (**b**) FTIR spectra, (**c**) XRD analysis, (**d**) thermogravimetric analysis (TGA), differential thermogravimetric (DTG) and differential scanning calorimetry (DSC), (**e**) nitrogen adsorption–desorption isotherm, (**f**) pore size distribution, and (**g**) magnetic hysteresis loops of magnetic MgO@BC.

The surface morphology of magnetic MgO@BC was observed using SEM (Figure 1a). Compared with raw crab shell biochar (Figure S3), the magnetic MgO@BC surface exhibited numerous granular structures with particles approximately 100 nm in diameter. EDS further confirmed the presence of Mg and Fe, indicating effective incorporation of the metal oxides. Figure S4 in the Supporting Information indicates that the composition ratio of Mg/Fe was 0.82.

The structural characteristics of magnetic MgO@BC was examined by FTIR (Figure 1b). A broad and intense absorption band centered at 3420 cm^−1^ was assigned to the stretching vibrations of surface –OH groups and residual –NH_2_ moieties, which are typically associated with adsorbed water and the biochar matrix. The band at 1628 cm^−1^ corresponded to the stretching vibration of C=O groups, indicating the presence of oxygen-containing functionalities. A distinct peak at 1420 cm^−1^ was attributed to the C–O stretching of CO_3_^2−^, mainly originating from naturally occurring calcium carbonate in the crab-shell precursor. The absorption near 1040 cm^−1^ was related to C–H stretching and bending vibrations. A narrow band at 874 cm^−1^ represented the out-of-plane bending vibration of CO_3_^2−^, further confirming carbonate species [23]. Finally, the low-frequency bands at 589 cm^−1^ and 485 cm^−1^ are assigned to the stretching vibrations of Fe–O–Fe and Mg–O, respectively, confirming the successful incorporation of magnetic iron oxide and magnesium oxide phases into the biochar framework [24,25].

Furthermore, the crystalline phases of magnetic MgO@BC were analyzed by XRD in Figure 1c. The diffraction pattern exhibited well-defined characteristic peaks of both MgO (PDF#00-045-0946) and Fe_3_O_4_ (PDF#00-028-0491), providing solid evidence for the successful introduction of these metal oxides into the biochar framework. The presence of MgO contributes abundant basic sites that can enhance electrostatic attraction and surface precipitation with heavy metal ions, while Fe_3_O_4_ not only imparts magnetic recoverability but also offers coordination sites for surface complexation. These structural features collectively demonstrate that the synthesis strategy effectively anchored MgO and Fe_3_O_4_ onto the biochar, thereby laying the foundation for its superior adsorption performance toward both organic dyes and heavy metal contaminants.

The thermal decomposition behavior of magnetic MgO@BC was systematically investigated using TGA, DTG, and DSC, as shown in Figure 1d. The TGA curve revealed a multi-stage weight loss profile. A minor weight loss (3–5%) was observed in this initial stage, primarily attributed to the loss of physically adsorbed water and volatile organic compounds. This was accompanied by a weak endothermic peak in the DSC curve, indicating an energy-absorbing dehydration process. The most significant weight loss (10–15%) occurred in the second stage. This phase corresponded to the thermal degradation of labile organic functional groups, such as proteins, chitin, and polysaccharides originating from the crab shell biochar. The DTG curve exhibited a sharp peak at around 400 °C, indicating the maximum decomposition rate. The relatively flat DSC signal in this range suggested a combination of endothermic and exothermic reactions, likely due to partial carbonization and release of volatiles. A gradual weight loss (25–30%) was recorded in this region, indicating further decomposition of more thermally stable organic matrices and possibly the breakdown of inorganic carbonates or transformation of metal oxides. The DSC curve showed a pronounced exothermic peak near 700–750 °C, suggesting intense carbon rearrangement or oxidation-like reactions, even under inert atmosphere, which was typical for the structural reorganization of carbon frameworks or the crystallization of inorganic phases (e.g., MgO or Fe_3_O_4_) At the end of the test (800 °C), the residual weight remained at approximately 45–50%, indicating the formation of a thermally stable carbonaceous and inorganic framework [26,27,28]. This suggests that the prepared magnetic MgO@BC sample possessed excellent thermal resistance, which was beneficial for its reuse and regeneration in environmental applications such as heavy metal or dye adsorption under high-temperature conditions.

The nitrogen adsorption–desorption isotherms and pore size distribution of magnetic MgO@BC were shown in Figure 1e,f. The isotherms in Figure 1e exhibited a typical type IV pattern with a pronounced hysteresis loop in the relative pressure range of 0.4–1.0, indicating a mesoporous structure according to the IUPAC classification [29]. The sharp increase in nitrogen uptake at high relative pressures further suggested the presence of mesopores along with a fraction of macropores. The BJH pore size distribution curve in Figure 1f revealed that the majority of pores fall within the 2–10 nm range, confirming that mesopores dominated the structure of magnetic MgO@BC. The BET analysis in Table 1 determined magnetic MgO@BC’s specific surface area to be 114.00 m^2^/g, a total pore volume (single-point adsorption) to be 0.30 cm^3^/g, and an average pore diameter to be 16.87 nm. These parameters were consistent with the characteristic of mesoporous materials. The mesoporous architecture facilitated the mass transfer and provided abundant accessible surface sites for Congo Red molecules and heavy metal ions during the adsorption process [30].

The magnetic behavior of magnetic MgO@BC was measured with a VSM at ambient temperature, and the resulting magnetization curve was shown in Figure 1g. The hysteresis loop revealed a typical S-shaped curve with negligible coercivity and remanence, indicating the superparamagnetic nature of the composite. The saturation magnetization of magnetic MgO@BC was found to be approximately 21 emu/g, indicating that magnetic Fe_3_O_4_ nanoparticles were successfully embedded in the biochar matrix. Such magnetic behavior offered advantageous for practical applications, enabling rapid separation of the adsorbent from aqueous solutions under an external magnetic field. The sufficient magnetization ensured effective magnetic responsiveness without compromising the material’s porous structure or adsorption capacity [31]. Overall, the VSM analysis confirms that magnetic MgO@BC possessed excellent magnetic recoverability, making it a promising candidate for environmental remediation applications.

### 3.2. In Single-Pollutant Systems

#### 3.2.1. Effects of Individual Factors on Adsorption Performance

##### Effect of Initial Pollutant Concentration

**Figure 2:**
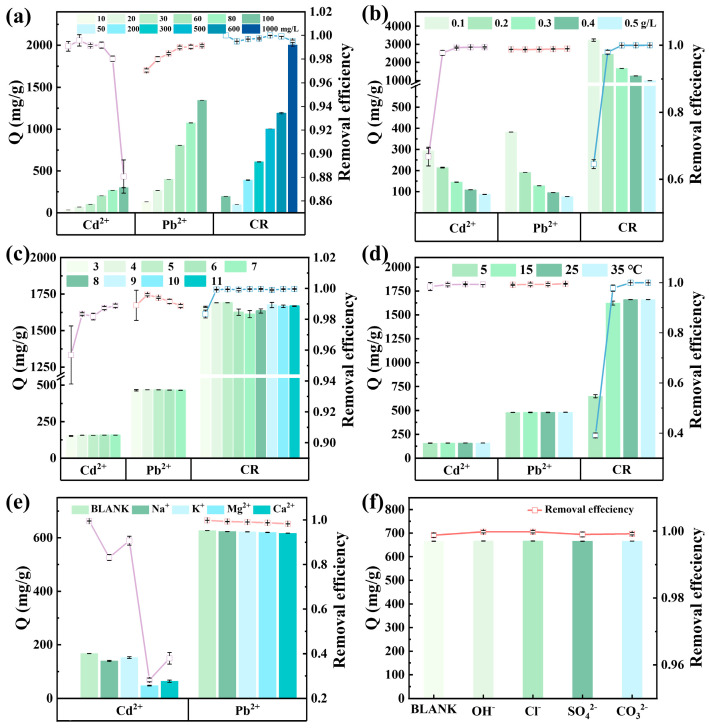
The effects of (**a**) initial pollutant concentration, (**b**) adsorbent dosage, (**c**) pH, and (**d**) temperature on the adsorption of Cd^2+^, Pb^2+^, and CR by magnetic MgO@BC. (**e**) The effects of various cations on the adsorption of Cd^2+^ and Pb^2+^. (**f**) The effects of various anions on the adsorption of CR.

The effect of initial pollutant concentration on adsorption performance is presented in Figure 2a. At an initial concentration of 10 mg/L, the adsorption capacity (Q) for Cd^2+^ was 33.87 mg/g. When the concentration increased to 100 mg/L, the adsorption capacity rose to 301.06 mg/g. However, when the initial concentration exceeded 80 mg/L, the removal efficiency dropped sharply from 98.02% to 88.07%, indicating rapid saturation of active sites under high-concentration conditions.

In contrast, Pb^2+^ exhibited a pronounced increase in Q from 131.57 mg/g at 10 mg/L to 1344.11 mg/g at 100 mg/L, while maintaining removal efficiencies consistently above 97%, reflecting a strong binding affinity and efficient utilization of adsorption sites even at elevated concentrations.

For CR, the Q value escalated from 97.84 mg/g at 50 mg/L to 2001.70 mg/g at 1000 mg/L, with removal efficiencies remaining nearly constant (>99%). This suggests that adsorption sites were not yet fully saturated, likely attributable to multiple adsorption mechanisms (e.g., electrostatic attraction and π–π interactions).

Overall, while increasing the initial pollutant concentration enhanced Q for all three contaminants, the impact on removal efficiency varied considerably. Cd^2+^ was highly susceptible to site saturation, Pb^2+^ sustained high efficiency, and CR displayed negligible sensitivity to concentration changes.

##### Effect of Adsorbent Dosage

As shown in Figure 2b, the adsorption behavior of Cd^2+^, Pb^2+^, and CR on magnetic MgO@BC was markedly influenced by adsorbent dosage. As the adsorbent dosage increased from 0.1 to 0.5 g/L, the total number of binding sites rose, causing the overall removal rate of Cd^2+^ and CR initially to increase and then level off. At an adsorbent dosage of 0.1 g/L, the maximum adsorption capacity of CR reached 3232.10 mg/g. Table S1 presents the reaction conditions corresponding to the maximum adsorption capacity, which was obtained from experimental data. While the removal efficiency of Pb^2+^ remained above 98.71% across the entire dosage range, indicating its exceptionally strong affinity and highly efficient site utilization [32].

The adsorption capacity Q for all three pollutants generally decreased due to the reduced average number of molecules captured per gram of adsorbent. At elevated dosages, particle aggregation and overlapping active sites may further reduce the accessible surface area, exacerbating the decline in Q. Considering both the removal efficiency and the potential redundancy of adsorption sites, the subsequent experiments in this study were conducted using the optimal adsorbent dosage determined for each respective pollutant. The experimentally identified optimal dosages were 0.3 g/L for Cd^2+^, 0.1 g/L for Pb^2+^, and 0.3 g/L for CR.

##### Effect of pH

The pH of the solution plays a key role in adsorption by affecting the adsorbent’s surface charge and the ionization of the adsorbate. The pH-dependent adsorption behaviors of Cd^2+^, Pb^2+^, and CR on magnetic MgO@BC are presented in Figure 2c. When pH > 7, high concentrations of OH^−^ in the solution can react with Cd^2+^ or Pb^2+^ to form Cd(OH)_2_ or Pb(OH)_2_ precipitates, interfering with removal rate calculations. Therefore, the effect of initial pH on the adsorption performance of magnetic MgO@BC for Cd^2+^ and Pb^2+^ solutions was investigated under a pH range of 3–7.

The removal efficiency of Cd^2+^ exceeded 95.69% within pH 3 to 7, with both efficiency and adsorption capacity (Q) increasing slightly with pH. At low pH conditions, competitive adsorption between H^+^ and Cd^2+^ suppressed surface co-precipitation, hindered the adsorption process. These effects diminished under near-neutral conditions, enhancing adsorption.

Figure 2c shows that at pH 3, the removal efficiency of Cd^2+^ reached 95.69%. A large amount of H^+^ in the solution competed with Cd^2+^ for adsorption sites. Simultaneously, the acidic functional groups on the surface of magnetic MgO@BC became positively charged due to protonation, exerting electrostatic repulsion on Cd^2+^. As the pH increased, the removal efficiency rose to 98.88%. This was attributed to the decrease in H^+^ concentration, resulting in weakened electrostatic repulsion and competitive effects, thereby significantly enhancing adsorption efficiency.

For Pb^2+^, a 98.87% removal rate was maintained within the pH range of 3–7, with a pronounced removal peak observed at pH 4. This reflected its strong affinity for the active sites and low pH sensitivity. When the pH was below 4, high concentrations of H^+^ competed with the active sites, limiting adsorption efficiency. As pH increased to 4, competitive effects significantly diminished. Concurrently, changes in Pb^2+^ speciation facilitated easier complexation with hydroxyl (**–**OH) and carboxyl (**–**COOH) groups on the surface, thereby enhancing adsorption capacity.

The adsorption capacity of magnetic MgO@BC toward CR was also studied within the pH range of 3–11. For CR, adsorption capacity and removal efficiency were nearly invariant with pH, maintaining approximately 100% removal efficiency. This indicates that adsorption was governed mainly by electrostatic attraction and π–π interactions, exhibiting insensitive to solution pH. Overall, magnetic MgO@BC exhibited outstanding adsorption performance with a broad pH adaptability [33].

##### Effect of Temperature

Figure 2d indicates that temperature variations (5–35 °C) induced only slight changes in Q and removal efficiency for Cd^2+^ and Pb^2+^, suggesting that their adsorption was largely temperature-independent. In contrast, CR adsorption increased significantly with temperature, implying a partially endothermic process. Elevated temperatures accelerated internal mass transfer within the biochar and enhanced accessibility of CR to internal adsorption sites [34,35].

##### Effect of Interfering Substances

The effect of different cations (Na^+^, K^+^, Mg^2+^ and Ca^2+^, 2500 mg/L) on the adsorption of Cd^2+^ and Pb^2+^ by magnetic MgO@BC was investigated. Figure 2e demonstrate that the introduction of cations had negligible impact on Pb^2+^ adsorption, with both adsorption capacity (Q) and removal efficiency remaining virtually unchanged compared to the blank. This result indicates that the high binding affinity and site specificity of magnetic MgO@BC towards Pb^2+^ may render it resistant to displacement by other cations. In contrast, Cd^2+^ adsorption exhibited pronounced sensitivity to co-existing cations, particularly Mg^2+^ and Ca^2+^, which caused substantial decreases in Q. The strong inhibitory effect of Mg^2+^ was attributed to its higher charge density and smaller ionic radius, which enhanced electrostatic screening and competitive occupation of negatively charged sites, thereby suppressing Cd^2+^ binding. Ca^2+^ produced a slightly weaker but still significant inhibitory effect via the same competition mechanism. While monovalent cations (Na^+^, K^+^) exerted minimal interference, suggesting weaker competitive or complexation interactions. Figure 2f shows that the Q and adsorption efficiency of CR on magnetic MgO@BC remained largely unchanged in the presence of different anions, indicating that CR adsorption was unaffected by ion competition.

Overall, these findings highlight that the adsorption of Pb^2+^ and CR is governed by high-affinity, site-specific interactions that are robust against ionic competition, whereas Cd^2+^ adsorption is more susceptible to ionic strength and divalent cation effects. This mechanistic distinction underscores the importance of considering ion composition in real wastewater applications, as competitive adsorption and surface-charge modulation significantly impact removal efficiency for weaker-binding contaminants [36,37].

#### 3.2.2. Adsorption Isotherm, Kinetic Characteristics and Adsorption Thermodynamics

##### Adsorption Isotherm

**Figure 3:**
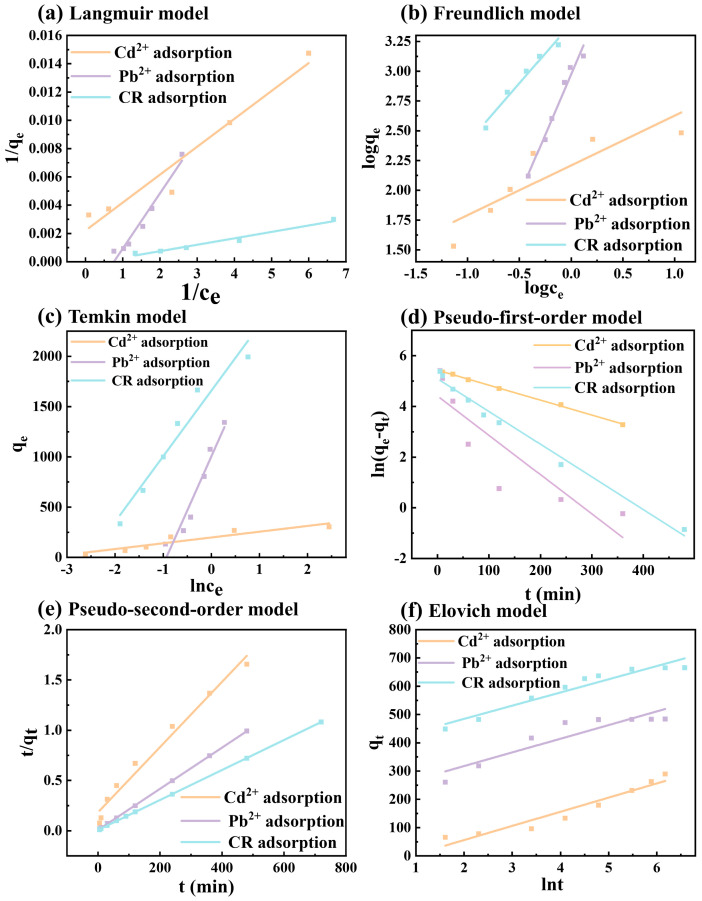
(**a**–**c**) Fitting of adsorption isotherms of Cd^2+^, Pb^2+^ and CR by magnetic MgO@BC. (**d**–**f**) Fitting of adsorption kinetics of Cd^2+^, Pb^2+^ and CR by magnetic MgO@BC.

The equilibrium adsorption behavior of Cd^2+^, Pb^2+^, and CR on magnetic MgO@BC was evaluated using the Langmuir, Freundlich, and Temkin isotherm models (Figure 3a–c and Table 2). The Langmuir model assumes a monolayer adsorption on a homogeneous surface with identical active sites and no interaction between adsorbed molecules, which can be expressed as Equation (1).

(1) $$q=\frac{q_{\max}K_{L}C_{e}}{1+K_{L}C_{e}}$$

where *q_e_* (mg/g) is the equilibrium adsorption capacity, *C_e_* (mg/L) is the equilibrium concentration, *q_max_* (mg/g) is the maximum monolayer capacity, and *K_L_* (L/mg) is the Langmuir constant.

The Freundlich model describes multilayer adsorption on heterogeneous surfaces and accounts for non-uniform distribution of adsorption heat [38], expressed as follows:

(2) $$q_{e}\;=\;K_{F}C_{e}^{{1/n}_{F}}$$

where *K_F_* is the adsorption capacity constant and n indicates the adsorption intensity. In the Freundlich model, 1/n*_F_* reflects surface heterogeneity and adsorption strength, with smaller values indicating a more heterogeneous surface and stronger adsorption.

The Temkin model considers the effect of adsorbate–adsorbent interactions and assumes that the heat of adsorption decreases linearly with surface coverage [39]. The expression is given in Equation (3).

(3) $$q_{e}\;=\;Bln(AC_{e})$$

where *A* (L/g) is the Temkin equilibrium binding constant and $B=\frac{RT}{b_{T}}$, with b*_T_* being the Temkin constant related to heat of adsorption.

Parameters of isotherms models for removal of Cd^2+^, Pb^2+^, and CR by magnetic MgO@BC were demonstrated in Table 2. For Cd^2+^, the Langmuir model showed a relatively good fit (R^2^ = 0.9961), yielding a maximum monolayer adsorption capacity (*q_max_*) of 244.67 mg/g and a relatively high affinity constant (*K_L_* = 2.2540). This suggests that Cd^2+^ adsorption may predominantly occur via monolayer coverage on homogeneous active sites. The weaker correlation with the Freundlich model (R^2^ = 0.9603) further supports the idea that uniform binding energies might dominate the Cd^2+^ adsorption process.

For Pb^2+^, the Langmuir model provided a better fit (R^2^ = 0.9916), with a maximum monolayer adsorption capacity (*q_max_*) of up to 968.10 mg/g and an affinity constant (*K_L_* = 0.4600). In contrast, the Freundlich model exhibited a lower correlation (R^2^ = 0.7737), indicating that Pb^2+^ adsorption may tend to follow a monolayer mechanism on homogeneous active sites [39,40].

For CR, the Freundlich model demonstrated a more prominent fit (R^2^ = 0.9963), with an adsorption constant *K_F_* = 662.64 and 1/n*_F_* = 0.7102 (ranging between 0 and 1). This implies that CR adsorption might take place on a heterogeneous surface with site energy distribution, involving multilayer adsorption, and the process could be favorable. While the Langmuir model also showed a high correlation (R^2^ = 0.9845), it was slightly lower than that of the Freundlich model, further supporting the possibility that CR adsorption may exhibit heterogeneous multilayer characteristics.

The Temkin model also showed strong fits for all three pollutants (R^2^ = 0.9960 for Cd^2+^; R^2^ = 0.9766 for Pb^2+^; R^2^ = 0.9963 for CR), suggesting that the heat of adsorption may decrease progressively with increasing surface coverage [41].

##### Kinetic Characteristics

To elucidate the adsorption mechanisms of Cd^2+^, Pb^2+^ and CR on magnetic MgO@BC, kinetic simulations were conducted using pseudo-first-order, pseudo-second-order, and Elovich models, respectively. The pseudo-first-order model assumes that the rate of occupancy of adsorption sites is proportional to the number of unoccupied sites (Equation (4)). The pseudo-second-order model describes chemisorption controlled by electron sharing or valence forces (Equation (5)). The Elovich model assumes adsorption on energetically heterogeneous surfaces (Equation (6)).

(4) $$ln(q_{e}\;-\;q_{t})\;=\;lnq_{e}\;-\;k_{1}t$$

(5) $$\frac{t}{q_{t}}=\frac{1}{k_{2}q_{e}^{2}}+\frac{t}{q_{e}}$$

where *q_t_* (mg/g) is the amount adsorbed at time *t*, *q_e_* is the equilibrium adsorption capacity, *k*_1_ (min^−1^) is the rate constant of the pseudo-first-order model, and k_2_ (g mg^−1^ min^−1^) is the rate constant of the pseudo-second-order model.

(6) $$q_{t}=\frac{1}{\beta }ln(\alpha \beta )+\frac{1}{\beta }lnt$$

where *α* (mg g^−1^ min^−1^) is the initial adsorption rate and *β* is the desorption constant (g/mg) [41,42].

Kinetic modeling results in Figure 3d–f and Table 3 show that the pseudo-first-order model provided a reasonably good fit for Cd^2+^ adsorption (R^2^ = 0.9984) and a reasonable fit for CR (R^2^ = 0.9832) but performed poorly for Pb^2+^ (R^2^ = 0.7436). In contrast, the pseudo-second-order model offered slightly better fits for Pb^2+^ (R^2^ = 0.9999) and CR (R^2^ = 1.0000), with predicted equilibrium adsorption capacities close to the experimental values (487.80 mg/g for Pb^2+^ and 671.14 mg/g for CR). However, it should be noted that these kinetic models are inherently empirical, and the difference in R^2^ values between the pseudo-first-order and pseudo-second-order models for Cd^2+^ is not significant, indicating that Cd^2+^ adsorption may be a complex multistep process. While the better fit of the pseudo-second-order model for Pb^2+^ and CR imply that chemisorption might play a certain dominant role in these systems. In addition, the Elovich model also provided reasonable fits for Cd^2+^ and CR, indicating that adsorption occurred on energetically heterogeneous surfaces, particularly for Cd^2+^ [43].

##### Adsorption Thermodynamics

The thermodynamic nature of Cd^2+^, Pb^2+^, and CR adsorption onto magnetic MgO@BC was investigated by analyzing the temperature-dependent equilibrium data using the Van’t Hoff equation. The adsorption thermodynamics were employed to evaluate the spontaneity, entropy change, and enthalpy change in the adsorption process, which can be expressed as Equations (7) and (8).

(7) $$△G^{0}=-RT\ln K_{d}$$

(8) $$\ln K_{d}=-\frac{△H^{0}}{R}\cdot \frac{1}{T}+\frac{△S^{0}}{R}$$

where Δ*G*^0^ (kJ/mol) is the standard Gibbs free energy change, Δ*H*^0^ (kJ/mol) is the standard enthalpy change, Δ*S*^0^ (kJ/(mol·K)) is the standard entropy change, *R* (8.314 J/(mol·K)) is the universal gas constant, and *T* (K) is the absolute temperature. The values of Δ*H*^0^ and Δ*S*^0^ were calculated from the slope and intercept of the linear plot of ln *K_d_* versus 1/*T*, respectively [44].

**Figure 4:**
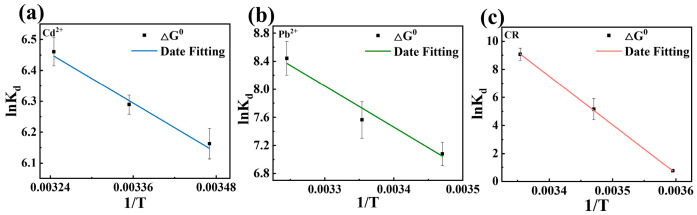
Fitting of adsorption thermodynamics of (**a**) Cd^2+^, (**b**) Pb^2+^ and (**c**) CR by magnetic MgO@BC.

The thermodynamic properties of Cd^2+^, Pb^2+^, and CR adsorption onto magnetic MgO@BC were analyzed by fitting the linear relationship between ln*K_d_* and 1/*T* (Figure 4a–c), and the corresponding thermodynamic parameters (Δ*G*^0^, Δ*H*^0^, Δ*S*^0^) were obtained (Table 4). As presented in Table 4, all Δ*G*^0^ values were negative and became more negative with rising temperature, suggesting that the adsorption of Cd^2+^, Pb^2+^, and CR was spontaneous and facilitated at higher temperatures. The positive Δ*S*^0^ values imply increased randomness at the solid–liquid interface, possibly due to solvent molecule release or heterogeneous active site exposure. Meanwhile, positive Δ*H*^0^ values indicate that the adsorption was an endothermic process, aligning with the temperature-dependent Δ*G*^0^ trend.

**Table 4:** Parameters of adsorption thermodynamics for removal of Cd^2+^, Pb^2+^, and CR by magnetic MgO@BC.

Pollutant	T(K)	ΔG^0^ (KJ/mol)	ΔS^0^ (KJ/mol-K)	ΔH^0^ (KJ/mol)
Cd^2+^	288.15	−14.7649	0.0894	11.0326
	298.15	−15.5901		
	308.15	−16.5524		
Pb^2+^	288.15	−16.9616	0.2279	48.7860
	298.15	−18.7522		
	308.15	−21.6254		
CR	278.15	−1.7549	1.0396	287.4006
	288.15	−12.3552		
	298.15	−22.5106		

### 3.3. Comparison with Other Biochar Adsorbents and Reusability Results

Integrating the isotherm and kinetic findings, Cd^2+^ adsorption was best characterized by monolayer chemisorption on relatively uniform active sites, as evidenced by its strong Langmuir fit and pseudo-first-order kinetics. In contrast, Pb^2+^ and CR adsorption were governed by heterogeneous surface interactions and variable binding energies, as indicated by their strong Freundlich and Temkin correlations, while still following pseudo-second-order kinetics [45].

Table 5 presents the comparison adsorption capacities of magnetic MgO@BC and other modified biochar materials for Cd^2+^, Pb^2+^ and CR. Magnetic MgO@BC exhibited greater adsorption capacity than other biomass adsorbents, indicating the significant potential of this highly efficient, recyclable adsorbent in dye wastewater treatment.

**Figure 5:**
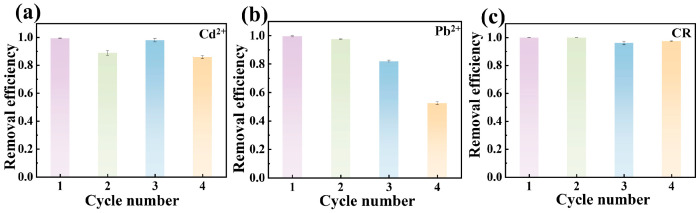
Reusability of magnetic MgO@BC after adsorption of (**a**) Cd^2+^, (**b**) Pb^2+^ and (**c**) CR.

The reusability of magnetic MgO@BC was evaluated through thermal regeneration at 700 °C under N_2_ (30 min, 10 °C/min heating/cooling). As shown in Figure 5a, for Cd^2+^, the removal efficiency remained 85.87% over four cycles. Interestingly, a partial recovery of Cd^2+^ removal efficiency was observed in the third cycle. This can be attributed to the progressive decomposition or transformation of residual deposits during repeated heating, reopening blocked pores and exposing new hydroxyl or oxide sites [36].

In contrast, Figure 5b shows that Pb^2+^ exhibited excellent reusability within the first two cycles but declined significantly thereafter. After the fourth cycle, the removal efficiency dropped to only 52.43%. This trend indicates that PbO, likely generated during the heat treatment process, may cause partial pore blocking and thus lead to a decrease in removal efficiency after multiple cycles [48].

Notably, CR exhibited the highest regeneration stability, maintaining 96.09% removal efficiency even after four cycles (Figure 5c). This stability was ascribed to the dominance of reversible π–π interaction and electrostatic forces, which can be largely restored after thermal treatment [49].

Overall, thermal regeneration was found to be more effective for organic pollutants (e.g., CR) than heavy metals (Cd^2+^, Pb^2+^). The irreversible complexation and precipitation of heavy metals reduced material’s reusability, though thermal activation in subsequent cycles could partially recover performance. In contrast, the adsorption of CR was mainly governed by reversible non-covalent interactions, enabling sustained adsorption capacity across multiple regeneration cycles.

### 3.4. In Binary-Pollutant Systems

In the single-component systems, magnetic MgO@BC exhibited strong adsorption capacities toward Cd^2+^, Pb^2+^, and the organic dye Congo Red (CR). To further evaluate its co-adsorption performance in binary systems, the adsorption capacity ratio (R_q_) was employed as a metric, as shown in Equation (7).

(9) R_q_ = q_b,i_/q_s,i_

where q_b,i_ is the adsorption capacity of component in binary-pollutant system, and q_s,i_ is the adsorption capacity in the corresponding single-component system. An R_q_ > 1 indicates a synergistic effect, R_q_ = 1 indicates no effect, and R_q_ < 1 reflects competitive inhibition [50]. The equilibrium concentrations of pollutants in the binary system are shown in Table S2.

**Figure 6:**
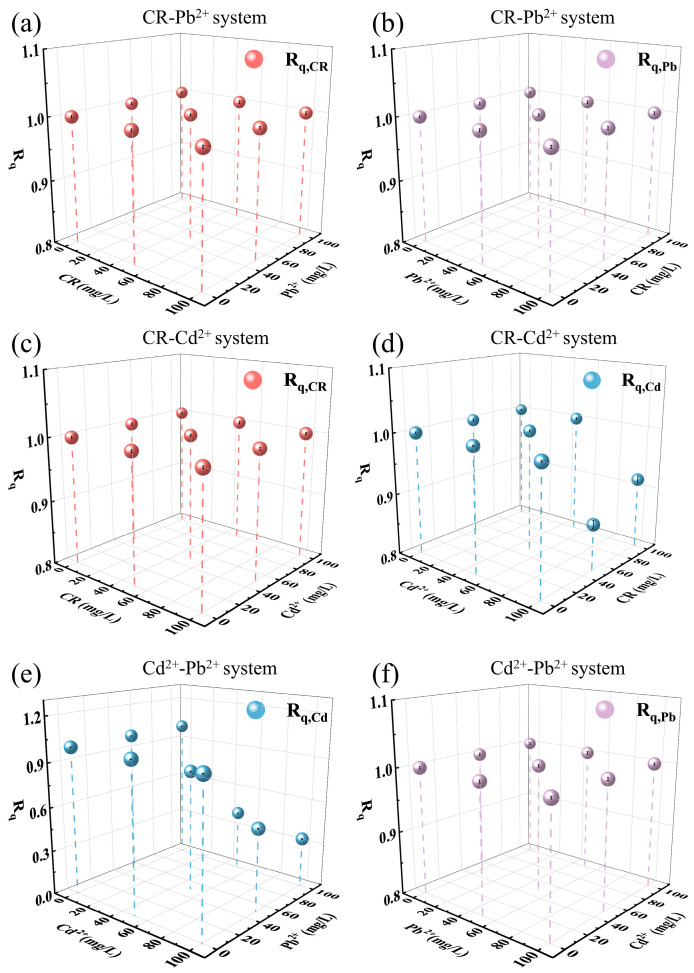
Simultaneous adsorption of Cd^2+^, Pb^2+^, and CR by magnetic MgO@BC in (**a**,**b**) CR–Pb^2+^ system, (**c**,**d**) CR–Cd^2+^ system, and (**e**,**f**) Cd^2+^–Pb^2+^ system.

In the CR–Pb^2+^ system (Figure 6a,b), both R_q,CR_ and R_q,Pb_ were approximately equal to 1, with R_q,Pb_ slightly decreasing at high CR concentrations. Pb^2+^ primarily combined with electronegative groups on the surface of magnetic MgO@BC via strong coordination bonds, while CR removal was driven by electrostatic attraction and π–π interactions with the carbonaceous matrix. Simultaneously, the coprecipitation of Pb^2+^ with carbonates in magnetic MgO@BC material made the adsorption process less susceptible to CR interference.

In systems where CR–Cd^2+^ coexist, Figure 6c shows that R_q,CR_ consistently approached 1, implying CR adsorption was nearly unaffected by coexisting Cd^2+^. Magnetic MgO@BC can completely remove CR from the mixed solution due to its superior adsorption capacity. Figure 6d indicates that R_q,Cd_ values remained approaching to 1 at lower initial Cd^2+^ concentrations (≤60 mg/L). However, at the higher Cd^2+^ concentration of 100 mg/L, CR exhibited a degree of inhibition on Cd^2+^ adsorption, with R_q,Cd_ values ranging from 0.87 to 1.00. This indicates that high CR concentrations occupied adsorption sites and competed for diffusion within pores, further limiting Cd^2+^ adsorption.

In the Cd^2+^–Pb^2+^ system (Figure 6e,f), a clear competitive hierarchy was observed. R_q,Pb_ was near 1, while R_q,Cd_ < 1, with the strongest inhibition occurring at higher Pb^2+^ levels. This selectivity was attributed to the higher affinity of Pb^2+^ for magnetic MgO@BC, arising from its lower hydration energy and greater electronegativity, which allowed Pb^2+^ to preferentially occupy adsorption sites [51].

### 3.5. Elucidation of Adsorption Mechanisms

#### 3.5.1. SEM and EDS Analysis of Magnetic MgO@BC After Adsorption

**Figure 7:**
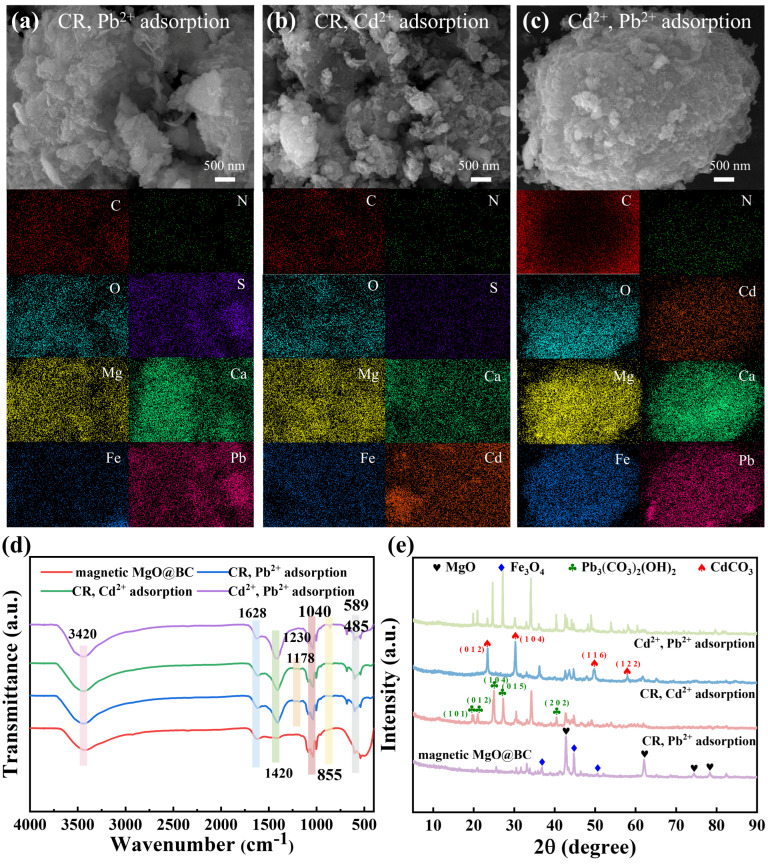
(**a**–**c**) The SEM images and the elemental mappings and the (**d**) FTIR spectra and (**e**) XRD spectra of magnetic MgO@BC after adsorption in binary-pollutant systems.

The surface morphology of magnetic MgO@BC after adsorption was characterized using SEM, and its elemental composition was further analyzed by EDS (Figure 7a–c). After adsorption, the EDS spectra clearly revealed the uniform distribution and concentrations of S (0.25%), Pb (1.97%) (Figure 7a), S (0.62%), Cd (4.26%) (Figure 7b), and Cd (0.27%), Pb (0.43%) (Figure 7c). This indicates that contaminants not only adsorbed onto the surface but also penetrated into the internal structure of magnetic MgO@BC [52].

#### 3.5.2. FTIR Analysis

To elucidate the changes in surface functional groups of magnetic MgO@BC, FTIR spectroscopy was conducted in Figure 7d. The stretching vibration band of surface hydroxyl groups (–OH) at 3420 cm^−1^ showed a slight decrease in intensity after adsorption, indicating that –OH groups participated in hydrogen bonding or surface complexation with the adsorbates [53]. After CR adsorption, two new peaks emerged at 1230 cm^−1^ and 1178 cm^−1^, corresponding to the asymmetric and symmetric S=O stretching vibrations of the sulfonate (–SO_3_^−^) groups in CR, thereby confirming the successful immobilization of CR onto the adsorbent surface. Following adsorption, the carbonate-related bands near 1420 cm^−1^ and 855 cm^−1^ exhibited a slight blue shift and enhanced intensity, suggesting that Pb^2+^ and Cd^2+^ may have formed carbonate complexes through a precipitation mechanism. Meanwhile, the bands at 589 cm^−1^ and 485 cm^−1^, assigned to the stretching vibrations of Fe–O–Fe and Mg–O, respectively, remained essentially unchanged before and after the reaction.

#### 3.5.3. XRD Analysis

A blank experiment with pure water treatment was conducted on magnetic MgO@BC under adsorption-like conditions. The XRD results in Figure S5 show that the diffraction peak profile of magnetic MgO@BC treated with pure water was basically similar to that of the original sample, indicating that no obvious structural damage or phase transformation of magnetic MgO@BC occurred under the tested conditions. After adsorption, new crystalline phases were observed in Figure 7e. For magnetic MgO@BC after adsorption in CR–Cd^2+^ system, distinct peaks of CdCO_3_ (PDF#00-042-1342) were detected, suggesting that Cd^2+^ was immobilized through carbonate precipitation [54]. For magnetic MgO@BC after adsorption in CR–Pb^2+^ system, characteristic peaks of Pb_3_(CO_3_)_2_(OH)_2_ (PDF#98-000-0248) were identified, indicating that Pb^2+^ was predominantly captured via basic lead carbonate formation. These stable precipitates not only account for the high removal efficiency of heavy metals but also explain the irreversible loss of active sites during regeneration due to their limited thermal decomposition. In the coexisting Cd^2+^–Pb^2+^ system, both CdCO_3_ and Pb_3_(CO_3_)_2_(OH)_2_ phases were observed, confirming that competitive adsorption and co-precipitation occurred simultaneously.

#### 3.5.4. Insights from XPS and Structural Analysis

**Figure 8:**
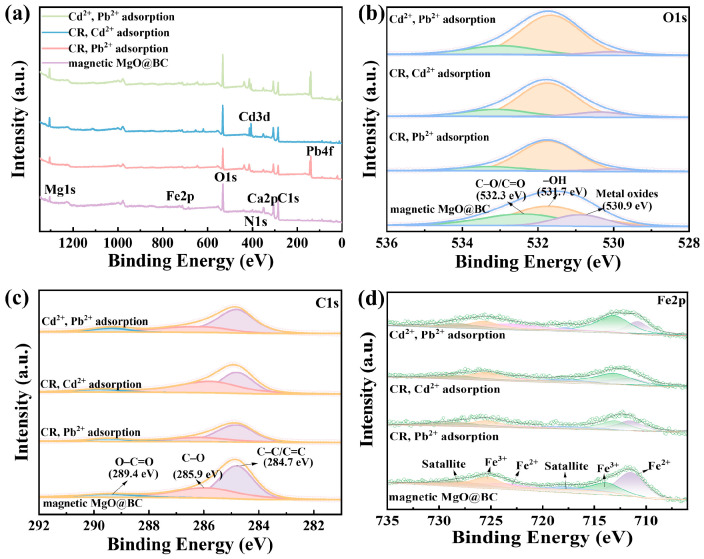
(**a**) XPS spectra, (**b**) C 1s spectra, (**c**) O 1s spectra and (**d**) Fe 2p spectra of magnetic MgO@BC after adsorption in binary-pollutant systems.

The XPS survey spectra (Figure 8a) reveal the dominant signals of Mg [55], Fe, C, O, and N, while additional peaks appearing after adsorption corresponded to Pb 4f (138–143 eV) and Cd 3d (404–411 eV), which was consistent with the successful capture and enrichment of heavy metals on the magnetic MgO@BC surface. The weakened Ca 2p peak after adsorption indicates that residual CaCO_3_ or carbonate groups originating from crab shell precursors may have been involved in the coprecipitation reaction with heavy metals [54,56].

High-resolution C 1s spectra in Figure 8b can be deconvoluted into three major components, including C–C/C=C (284.6–284.8 eV), C–O (285.6–286.0 eV), and O–C=O (289.0–289.6 eV) [57]. Compared with pristine magnetic MgO@BC, the adsorbed samples displayed the following characteristics: (i) the C–C/C=C bond at 284.7 eV remained the dominant peak, suggesting a stable carbon skeleton; (ii) the slight binding energy shifts in C–O and O–C=O suggested that oxygenated groups (–OH, –COOH) may have been involved through deprotonation and metal coordination; (iii) the intensity of the O–C=O (289.4 eV) significantly enhanced, especially in Pb-containing samples, which was consistent with the possible formation of M–O–C linkages or covalent coordination/precipitation in the form of metal–carboxylates or carbonates. These results indicate that surface oxygenated groups (–COOH, C=O, C–O) likely played an important role in the adsorption process via deprotonation and coordination exchange, potentially leading to forming stable metal–organic interfaces [52].

Figure 8c demonstrates that the O 1s spectra can be fitted to lattice oxygen in metal oxides (M–O, 530.8–531.0 eV), hydroxyl/defect oxygen (–OH, 531.7 eV), and organic or carbonate oxygen (C–O/C=O, 532.2–532.5 eV). The peak intensity of metal oxides decreased after adsorption, suggesting that ion exchange interactions may have occurred between Pb^2+^, Cd^2+^ and magnetic MgO@BC. The relative increase in –OH groups implies that strong surface complexation may have been involved during the adsorption process, particularly in samples containing Cd^2+^. The C–O and C=O bonds were weakened and shifted after adsorption, indicating that these functional groups may have contributed to the adsorption process of pollutants. Possible underlying mechanisms may include electrostatic attraction, micro-precipitation, pore filling, and diffusion [58,59].

High-resolution Fe 2p XPS spectra of magnetic MgO@BC and adsorbate-loaded samples were deconvoluted to probe the oxidation states and interfacial changes (Figure 8d). The pristine magnetic MgO@BC showed characteristic Fe 2p_3/2_ components at 711.4 eV and 713.9 eV, corresponding to Fe^2+^ and Fe^3+^. Characteristic Fe 2p_1/2_ peaks were observed at 723.1 eV and 725.2 eV, corresponding to Fe^2+^ and Fe^3+^, respectively. Two weak satellite peaks appeared at 717.8 eV and 728.2 eV, further confirming the presence of magnetite (Fe_3_O_4_) in the composite material. After adsorption, systematic spectral changes were observed. Samples loaded with Pb^2+^ (CR–Pb^2+^ system) exhibited an increased Fe^3+^/Fe^2+^ ratio and a slight positive shift in Fe 2p peaks, suggesting a possible local decrease in electron density around Fe atoms, which may be related to strong Pb–O coordination or partial surface oxidation potentially induced by Pb^2+^ binding. In contrast, Cd^2+^-containing samples (CR–Cd^2+^ and Cd^2+^–Pb^2+^ systems) showed smaller peak shifts but overall reduced Fe signal intensity, which was consistent with possible surface coverage by adsorbed species. Together with complementary O 1s and C 1s data, these trends suggest that metal adsorption on magnetic MgO@BC may have proceeded primarily via inner-sphere surface complexation with oxygenated functional groups (–OH, C=O, –COO^−^), along with potential partial surface precipitation. The persistence of mixed-valence Fe signals after adsorption confirmed the structural stability of the magnetic phase, which preserved the material’s magnetic recoverability while providing metal-binding active sites.

#### 3.5.5. Adsorption Mechanism

The combined XRD and XPS analyses provided deeper insight into the adsorption mechanisms of magnetic MgO@BC toward different pollutants. XRD patterns revealed the formation of stable precipitates such as CdCO_3_ and Pb_3_(CO_3_)_2_(OH)_2_ after heavy metal adsorption. This observation was consistent with the binding energy shifts in the XPS spectra, suggesting that Cd^2+^ and Pb^2+^ may have been immobilized not only through surface complexation but also possibly via precipitation/co-precipitation processes. In contrast, no new crystalline phases were detected in CR-loaded samples by XRD, but significant shifts in the C 1s and O 1s peaks were observed in XPS. This suggests that the adsorption of CR may have mainly involved π–π interactions and electrostatic attraction. These non-covalent interactions were largely reversible and can be restored during high-temperature treatment, thereby ensuring superior regeneration stability.

**Figure 9:**
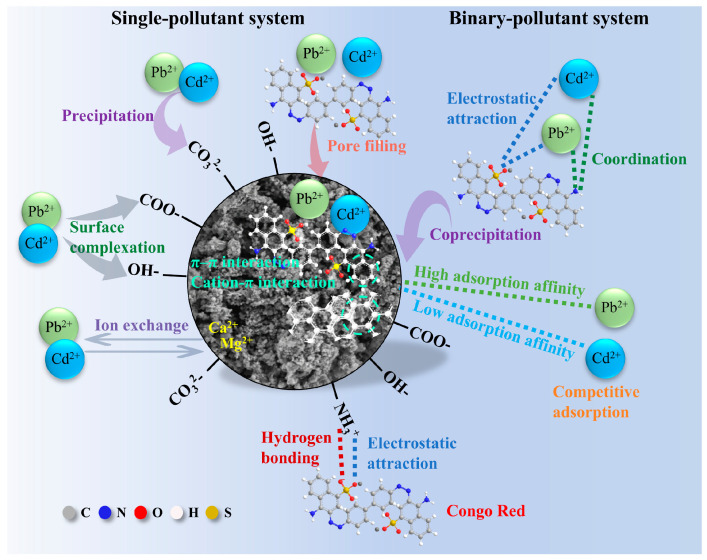
Schematic illustration of the adsorption mechanisms of magnetic MgO@BC in single-pollutant system and binary-pollutant system.

In summary, the adsorption mechanism of magnetic MgO@BC can be described as a multi-channel synergistic process (Figure 9). In single-pollutant systems, for CR, it is likely that (i) π–π interactions existed between CR’s aromatic rings and the biochar’s graphitic domains, and (ii) electrostatic attraction and hydrogen bonding interactions occurred between CR’s functional groups (–SO_3_^−^, –NH) and surface hydroxyls [60]. For metal ions, possible mechanisms include (i) cation–π interactions between Pb^2+^ and Cd^2+^ and the biochar’s graphitic domains, (ii) potential ion exchange of Pb^2+^ and Cd^2+^ with Ca^2+^ and Mg^2+^ within biochar, (iii) inner-sphere complexation of Pb^2+^/Cd^2+^ with deprotonated oxygen-containing groups (–COOH, –OH, C=O), and (iv) carbonate/hydroxide co-precipitation. Table S3 in the Supporting Information shows that the solution pH rose to approximately 7.6 after the reaction, indicating partial precipitation of Pb^2+^ and Cd^2+^. However, the pH changes were not significant enough to induce substantial bulk precipitation of metal ions. When surface groups approached saturation, contaminants may enter the porous structure via pore filling and diffusion, a process that may act as the main factor during the internal diffusion stage.

Notably, in binary-pollutant systems, Pb^2+^ exhibited stronger affinity for high-energy binding sites and may have induced carbonate/oxide immobilization, whereas Cd^2+^ was mainly stabilized by coordination with hydroxyl/carboxyl groups and might be more susceptible to competitive inhibition [61,62,63]. These results suggest that the adsorption process was likely governed by π–π interactions, electrostatic attraction, hydrogen bonding for CR and surface coordination, ion exchange, coprecipitation for heavy metals (Pb^2+^, Cd^2+^). Therefore, in coexisting systems, CR and Pb^2+^ remained largely unaffected, while high concentrations of CR or Pb^2+^ may have exerted some influence on Cd^2+^ adsorption, particularly in the Cd^2+^–Pb^2+^ system.

## 4. Conclusions

In this study, crab shell waste was converted into a functional magnetic biochar composite via wet impregnation and high-temperature carbonization. The prepared magnetic MgO@BC exhibited a porous structure, rich surface functionalities, and excellent magnetic separability, which may facilitate efficient adsorption and convenient recovery. Systematic batch experiments demonstrated that magnetic MgO@BC achieved high adsorption capacities and removal efficiencies for Cd^2+^, Pb^2+^, and CR. Thermodynamic analyses suggested that the adsorption was spontaneous, endothermic. Regeneration tests revealed superior reusability for CR and Cd^2+^, and moderate reusability for Pb^2+^. Binary-pollutant system experiments further emphasized the competitive or synergistic effects among coexisting pollutants, with adsorption likely dominated by π–π interactions, electrostatic attraction, hydrogen bonding, surface complexation, and precipitation. Overall, this work highlights the dual applicability of magnetic MgO@BC for removing both organic dyes and heavy metals, providing a sustainable strategy for transforming biomass waste into efficient multifunctional adsorbents for wastewater remediation.

Although the prepared magnetic MgO@BC exhibits excellent adsorption capacities for organic dyes and heavy metals, it still presents certain limitations. Future research will focus on expanding to binary and multi-component pollution systems to better simulate the complex environment of actual wastewater. For instance, the influence of key environmental factors such as pH, temperature, and ionic strength will be thoroughly investigated. By integrating adsorption isotherm, kinetic, and thermodynamic fitting analyses, the competitive adsorption mechanisms and interaction patterns among different pollutants will be elucidated. In addition, the regeneration performance of magnetic MgO@BC after adsorption saturation in binary systems will be systematically evaluated, with a focus on exploring the desorption behavior of pollutants and the recovery degree of material structure and active sites during the regeneration process. From a practical application perspective, the thermal regeneration at 700 °C used in this study still has certain limitations in terms of energy consumption and operational cost, which may hinder its large-scale continuous application. Future research could focus on exploring milder, more energy-efficient, and easier-to-operate regeneration strategies to further improve the economic feasibility and engineering scalability of the material. This work is expected to provide a theoretical basis and technical support for the cyclic application of magnetic MgO@BC in actual wastewater treatment. Through the aforementioned research, it is anticipated that the adaptability and practicality of magnetic MgO@BC in complex pollution systems will be comprehensively improved, advancing its progress towards engineering application.

## Figures and Tables

**Table 1:** The N_2_ adsorption–desorption analysis of the magnetic MgO@BC.

BET Surface Area	BJH Adsorption Average Pore Diameter (4 V/A)	a Total Pore Volume (Single-Point Adsorption)
114.00 m^2^/g	16.87 nm	0.30 cm^3^/g

**Table 2:** Parameters of isotherms models for removal of Cd^2+^, Pb^2+^, and CR by magnetic MgO@BC.

Pollutant	Langmuir Model		Freundlich Model		Temkin	
Cd^2+^	q_max_ (mg/g)	244.6713 ± 6.7127	K_F_	77.6283 ± 2.8491	A_T_	37.9887 ± 1.2065
	K_L_	2.2540 ± 0.0718	1/n_F_	0.3135 ± 0.0139	B	33.9872 ± 1.0770
	R^2^	0.9961	R^2^	0.9603	R^2^	0.9960
Pb^2+^	q_max_ (mg/g)	968.1040 ± 40.6991	K_F_	251.6981 ± 8.7015	A_T_	5.6058 ± 0.5531
	K_L_	0.4600 ± 0.0253	1/n_F_	0.3138 ± 0.0449	B	172.5711 ± 13.3292
	R^2^	0.9916	R^2^	0.7737	R^2^	0.9766
CR	q_max_ (mg/g)	4549.7617 ± 1107.2830	K_F_	662.6410 ± 1.4061	A_T_	2.6896 ± 0.1284
	K_L_	0.1599 ± 0.0622	1/n_F_	0.7102 ± 0.0017	B	755.2946 ± 22.9278
	R^2^	0.9845	R^2^	0.9963	R^2^	0.9963

**Table 3:** Parameters of kinetic model for removal of Cd^2+^, Pb^2+^, and CR by magnetic MgO@BC.

Pollutant	Pseudo-First-Order Model		Pseudo-Second-Order Model		Elovich	
Cd^2+^	q_e_ (mg/g)	227.8711	q_e_ (mg/g)	308.6420	α	20.7509
	k_1_	0.0026	k_2_	0.0001	β	0.0200
	R^2^	0.9984	R^2^	0.9751	R^2^	0.9203
Pb^2+^	q_e_ (mg/g)	82.4655	q_e_ (mg/g)	487.8049	α	4681.5950
	k_1_	0.0067	k_2_	0.0005	β	0.0207
	R^2^	0.7436	R^2^	0.9999	R^2^	0.8263
CR	q_e_ (mg/g)	162.7736	q_e_ (mg/g)	671.1409	α	1062.9921
	k_1_	0.0056	k_2_	0.0003	β	0.0026
	R^2^	0.9832	R^2^	1.0000	R^2^	0.9255

**Table 5:** Comparison of the adsorption performance of the modified biochar for Cd^2+^, Pb^2+^, and CR with literature reports.

Material (Brief)	Conditions (T, pH)	Pollutant	Reported q_max_ (mg/g)	BET Surface Area (m^2^/g)	Reference
Activated biochar (ABHC)	300.15 K; pH = 5.4	Congo Red (CR)	114.8 (Langmuir)	124.15	[35]
Phosphate rock-magnetic biochar (PR-MCLB)	298.15 K; pH: 5.0	Pb^2+^, Cd^2+^	Pb: 451.24 Cd: 120.87	13.66	[46]
MgO-containing magnetic composite biochar (MBC)	303.00 K; pH: 6.0	Pb^2+^	253.6	42.60	[31]
CTAB-modified orange peel biochar (NOBC)	298.15 K; None	Congo Red (CR)	609.8 (Langmuir)	618.44	[47]
Magnetic crab-shell biochar loaded with magnesium oxide (magnetic MgO@BC)	298.15 K; pH: CR: 9.68 Pb: 5.39 Cd: 5.93	Congo Red (CR) Pb^2+^, Cd^2+^	CR: 3232.10 Pb: 1344.11 Cd: 301.06	114.00	This study

## Data Availability

The original contributions presented in this study are included in the article/Supplementary Material. Further inquiries can be directed to the corresponding authors.

## Supplementary Materials

The following supporting information can be downloaded at: https://www.mdpi.com/article/10.3390/nano16030214/s1, Figure S1: Exploration of Fe-Mg impregnation ratio and calcination temperature. (a) 0.08 M MgCl_2_·6H_2_O with 0.02 M FeCl_2_·4H_2_O, and (b) 0.04 M MgCl_2_·6H_2_O with 0.02 M FeCl_2_·4H_2_O calcined at 600 °C, 700 °C and 800 °C; Figure S2: Removal efficiencies of BC, magnetic BC, and magnetic MgO@BC for Cd^2+^, Pb^2+^, and CR; Figure S3: The SEM images and the elemental mappings of BC; Figure S4: Mg and Fe composition in magnetic MgO@BC; Figure S5: The XRD patterns of magnetic MgO@BC and the blank magnetic MgO@BC; Table S1: Reaction conditions corresponding to maximum adsorption capacity; Table S2: The equilibrium concentrations of pollutants in the binary system; Table S3: pH values of pollutant solutions before and after the reaction.
