# Supplementary material for: MgO-Loaded Magnetic Crab Shell-Derived Biochar for Efficient Synergistic Adsorption of Heavy Metals and Dye: Characterization, Adsorption Performance and Mechanistic Study
Source: Nanomaterials (Basel). 2026 Feb 6;16(3):214. doi: 10.3390/nano16030214 (PMC12899160; doi:10.3390/nano16030214)
Supplement: Supplementary file 1 [file nanomaterials-16-00214-s001.zip › nanomaterials-4085903-supplementary.pdf]

Supporting Information

MgO-loaded magnetic crab shell-derived biochar for efficient synergistic adsorption of heavy metals and dye: characterization, adsorption performance and mechanistic study

Yangyi Du [a], Si Wu [a,b*], Tao Feng [a,b*], Wenxue Jiang [a]

[a] College of Resources and Environmental Engineering, Wuhan University of Science and Technology, Wuhan, 430081, China
[b] Hubei Key Laboratory for Efficient Utilization and Agglomeration of Metallurgic Mineral Resources. Wuhan University of Science and Technology, Wuhan, 430081, China

***Corresponding Authors**
E-mail: siwu@wust.edu.cn (S. Wu)
E-mail: fengtaowhu@163.com (T. Feng)

1. Exploration of material preparation

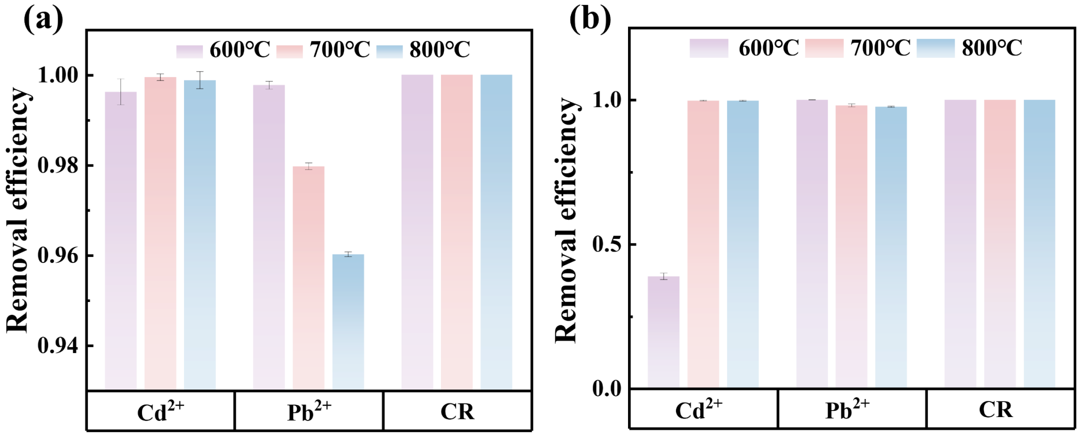

Figure S1. Exploration of Fe-Mg impregnation ratio and calcination temperature. (a) 0.08 M $MgCl_2 \cdot 6H_2O$ with 0.02 M $FeCl_2 \cdot 4H_2O$, and (b) 0.04 M $MgCl_2 \cdot 6H_2O$ with 0.02 M $FeCl_2 \cdot 4H_2O$ calcined at 600 °C, 700 °C and 800 °C.

Figure S1 explored the effects of Fe-Mg impregnation ratio and calcination temperature on the removal efficiencies of Cd^{2+}, Pb^{2+} and Congo Red (CR). The materials were prepared with two different impregnation ratios: 0.08 M $MgCl_2 \cdot 6H_2O$ with 0.02 M $FeCl_2 \cdot 4H_2O$, and 0.04 M $MgCl_2 \cdot 6H_2O$ with 0.02 M $FeCl_2 \cdot 4H_2O$. Samples were calcined at 600 °C, 700 °C and 800 °C, respectively. All experiments were conducted in triplicate and performed in a thermostatic shaker at 25 °C and 180 rpm. In the adsorption experiments, the counter-ion for both Pb^{2+} and Cd^{2+} was nitrate (NO_3^-),

and all contaminant concentrations were fixed at 50 mg/L with an adsorbent dosage of 0.2 g/L.

The results in Figure S1 show that although the 0.08 M $MgCl_2 \cdot 6H_2O$ loading produced slightly higher Cd^{2+} removal, it resulted in lower Pb^{2+} removal and weaker magnetic response, which was critical for subsequent solid–liquid separation. Taking both magnetic properties and overall removal performance into account, the impregnation ratio of 0.04 M $MgCl_2 \cdot 6H_2O$ with 0.02 M $FeCl_2 \cdot 4H_2O$ and a calcination temperature of 700 °C were selected as the optimal preparation condition.

2. Comparison of the adsorption performance between pure crab shell biochar (BC), magnetic BC, and magnetic MgO@BC

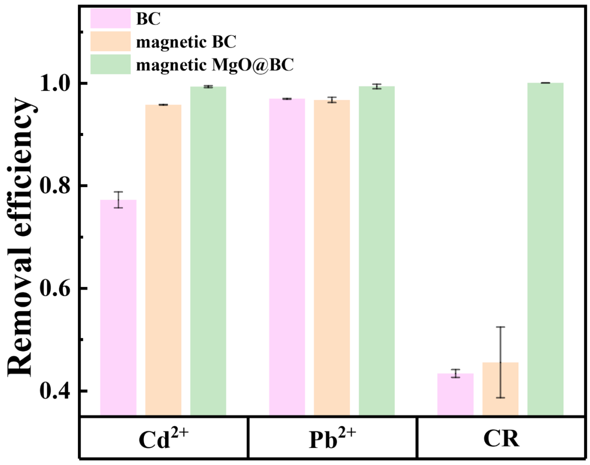

Figure S2. Removal efficiencies of BC, magnetic BC, and magnetic MgO@BC for Cd^{2+}, Pb^{2+}, and CR

Figure S2 shows the removal efficiencies of BC, magnetic BC, and magnetic MgO@BC toward Cd^{2+}, Pb^{2+}, and CR. The experiments were tested under the following conditions: Cd^{2+} at 50 mg/L with a dosage of 0.3 g/L, Pb^{2+} at 50 mg/L with a dosage of 0.1 g/L, and CR at 500 mg/L with a dosage of 0.3 g/L. All experiments were conducted in triplicate and performed in a thermostatic shaker at 25 °C and 180 rpm.

The results in Figure S2 demonstrate that magnetic MgO@BC exhibited a removal efficiency close to 1.0 for all three pollutants, making it the best-performing material overall. Magnetic BC also showed relatively high removal efficiencies for Cd^{2+} and Pb^{2+}, but its efficiency for CR was notably lower. The removal efficiency of BC for CR was only approximately 43.27 %, and its removal effects on Cd^{2+} and Pb^{2+} were also weaker than those of the other two materials. Overall, MgO modification can effectively enhance the removal capacity of the materials, particularly for CR. Moreover, the combination of MgO and magnetic modification enables the material to maintain a high removal rate while allowing for convenient recovery via its magnetic properties.

3. SEM and EDS analysis

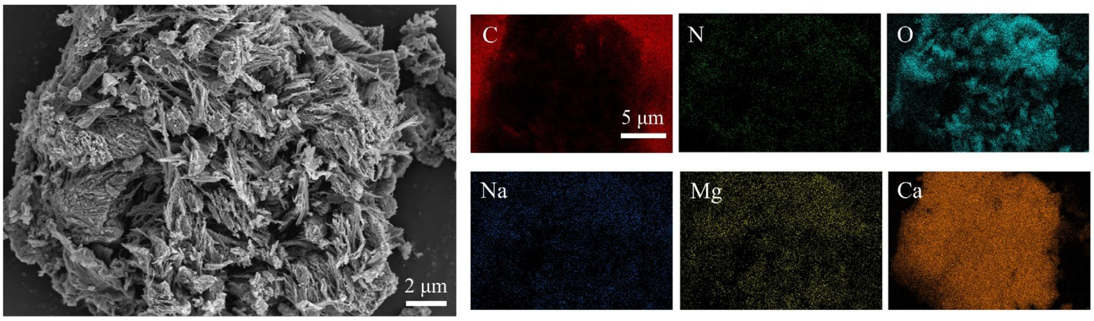

Figure S3. The SEM images and the elemental mappings of BC.

The SEM image of the raw crab shell biochar (BC) (Figure S3) revealed a rough and hierarchically porous surface, composed of interconnected micro- and macropores. EDS analysis confirmed the primary elements as calcium (Ca), oxygen (O), and carbon (C), consistent with the presence of $CaCO_3$ and the chitin matrix. While trace amounts of nitrogen (N) originated from chitin's amino groups.

4. Reaction conditions corresponding to maximum adsorption capacity

Table S1. Reaction conditions corresponding to maximum adsorption capacity.

Pollutant	Maximum Adsorption Capacity (q_{max})/(mg/g)	Adsorbent Dosage/(g/L)	Reaction Temperature/(℃)	pH	Data Type
Cd^{2+}	301.06	0.3	25	5.93	Experimental data
Pb^{2+}	1344.11	0.1	25	5.39	Experimental data
CR	3232.10	0.1	25	9.68	Experimental data

5. Mg and Fe composition in magnetic MgO@BC

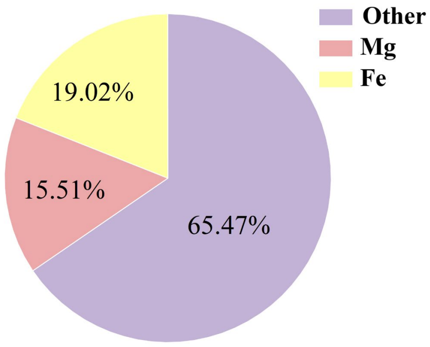

Figure S4. Mg and Fe composition in magnetic MgO@BC.

6. The equilibrium concentrations of pollutants in the binary system

Table S2. The equilibrium concentrations of pollutants in the binary system.

System	Pollutant concentration ratio (mg/L)	q_1(mg/g)	q_2(mg/g)
$CR–Pb^{2+}$	$C_{CR}:C_{Pb2+}$=50:50	q_{CR}=166.28±0.0000	q_{Pb2+}=165.48±0.1931
	$C_{CR}:C_{Pb2+}$=50:100	q_{CR}=166.28±0.0000	q_{Pb2+}=331.98±0.0644
	$C_{CR}:C_{Pb2+}$=100:50	q_{CR}=332.95±0.0000	q_{Pb2+}=165.48±0.1179
	$C_{CR}:C_{Pb2+}$=100:100	q_{CR}=332.95±0.0000	q_{Pb2+}=331.62±0.0432
$CR–Cd^{2+}$	$C_{CR}:C_{Cd2+}$=50:50	q_{CR}=166.28±0.0000	q_{Cd2+}=165.32±0.0040
	$C_{CR}:C_{Cd2+}$=50:100	q_{CR}=166.28±0.0000	q_{Cd2+}=262.44±1.9970
	$C_{CR}:C_{Cd2+}$=100:50	q_{CR}=332.95±0.0000	q_{Cd2+}=165.32±0.1372
	$C_{CR}:C_{Cd2+}$=100:100	q_{CR}=332.95±0.0000	q_{Cd2+}=267.91±4.8313
$Cd^{2+}–Pb^{2+}$	$C_{Cd2+}:C_{Pb2+}$=50:50	q_{Cd2+}=138.50±5.8025	q_{Pb2+}=165.80±0.1105
	$C_{Cd2+}:C_{Pb2+}$=50:100	q_{Cd2+}=66.36±13.6809	q_{Pb2+}=331.06±0.0407
	$C_{Cd2+}:C_{Pb2+}$=100:50	q_{Cd2+}=167.91±1.9825	q_{Pb2+}=165.25±0.1029
	$C_{Cd2+}:C_{Pb2+}$=100:100	q_{Cd2+}=103.60±10.4584	q_{Pb2+}=332.03±0.2168

7. pH values of pollutant solutions before and after the reaction

Table S3. pH values of pollutant solutions before and after the reaction.

Pollutant	Adsorbent Dosage/(g/L)	Reaction Temperature/(℃)	pH before the reaction	pH after the reaction
Cd^{2+}	0.3	25	5.93	7.64
Pb^{2+}	0.1	25	5.39	7.66
CR	0.1	25	9.68	10.17

8. Stability test of the material in pure water under adsorption-like conditions

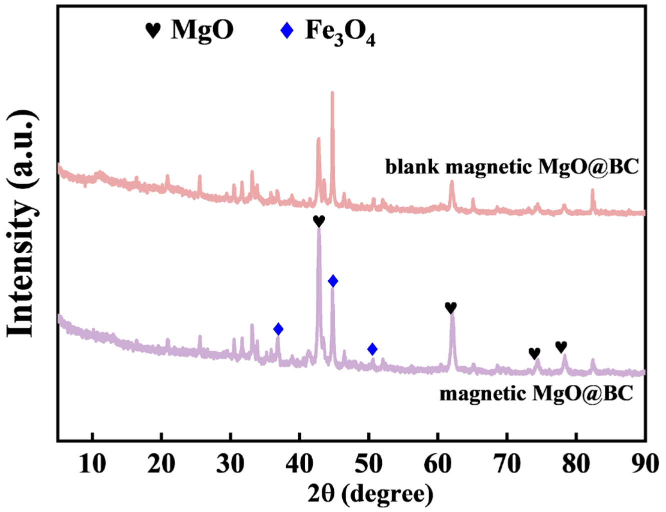

Figure S5. The XRD patterns of magnetic MgO@BC and the blank magnetic MgO@BC
